# Spatial variation in a top marine predator’s diet at two regionally distinct sites

**DOI:** 10.1371/journal.pone.0209032

**Published:** 2019-01-02

**Authors:** Martha Gosch, Michelle Cronin, Emer Rogan, William Hunt, Cian Luck, Mark Jessopp

**Affiliations:** 1 MaREI Centre, Environmental Research Institute, University College Cork, Cork, Ireland; 2 School of Biological, Earth & Environmental Sciences, University College Cork, Cork, Ireland; Department of Agriculture and Water Resources, AUSTRALIA

## Abstract

In ecological studies it is often assumed that predator foraging strategies and resource use are geographically and seasonally homogeneous, resulting in relatively static trophic relationships. However, certain centrally placed foragers (e.g. seals) often have terrestrial sites for breeding, resting, and moulting that are geographically distinct, and associated with different habitat types. Therefore, accurate estimations of predator diet at relevant spatial and temporal scales are key to understanding energetic requirements, predator-prey interactions and ecosystem structure. We investigate geographic variation in the diet of grey seals (*Halichoerus grypus*), a relatively abundant and widely distributed central place forager, to provide insights into geographic variation in resource use. Prey composition was identified using scat samples collected over concurrent timescales and a multivariate approach was used to analyse diet from two contrasting habitats. Regional differences in prey assemblages occurred within all years (2011–2013) and all seasons (ANOSIM, all p<0.05), apart from in winter. Telemetry data were used to identify core foraging areas and habitats most likely associated with scat samples collected at the two haul-out sites. Regional differences in the diet appear to reflect regional differences in the physical habitat features, with seals foraging in deeper waters over sandy substrates showing a higher prevalence of pelagic and bentho-pelagic prey species such as blue whiting and sandeels. Conversely, seals foraging in comparatively shallow waters had a greater contribution of demersal and groundfish species such as cephalopods and flatfish in their diet. We suggest that shallower waters enable seals to spend more time foraging along the benthos while remaining within aerobic dive limits, resulting in more benthic species in the diet. In contrast, the diet of seals hauled-out in areas adjacent to deeper waters indicates that either seals engage in a more pelagic foraging strategy, or that seals can spend less time at the benthos, resulting in comparatively more pelagic prey recovered in the diet. The substantial differences in prey assemblages over a small spatial scale (<300 km) demonstrates the importance of using regionally-specific diet information in ecosystem-based models to better account for different trophic interactions.

## Introduction

Foraging is one of the most important components of individuals’ reproductive success and survival [1]. A key behaviour associated with foraging includes movement, a prerequisite enabling predators to locate, pursue and capture prey [2]. In free-ranging marine mammals, the energetic costs required during foraging and subsequent prey digestion and assimilation are high [3, 4]. Animals with high-energy requirements have the potential to considerably impact local prey populations and often play important roles in the structure and functioning of communities [5, 6]. Equally, prey behaviour and physical habitat structure may also influence predator foraging behaviour [7]. Dynamic physical components of habitat such as tides and upwelling zones, and spatial complexity within the marine environment, dictates the structure of multispecies communities. Additionally, species interactions within ecosystems cause community structure changes seasonally, annually and regionally [8, 9]. To understand how top predators and their populations respond to changing ecological and environmental conditions, and to comprehend their functional roles in the marine ecosystem, information on foraging ecology, including diet, is necessary [10–12].

The grey seal (*Halichoerus grypus*, Fabricius, 1791) is considered a central place forager and a top predator within certain areas of the northeast Atlantic [13]. Despite displaying a high degree of site fidelity, seals move regularly between colonies, with distances from haul-out sites to foraging grounds differing regionally [14]. While seals can remain at sea for extended periods [15, 16, 17], foraging habitat selection typically occurs within close range of haul-out sites [13, 16–18]. This suggests that seal diet composition relates to prey availability and abundance surrounding the haul-out region [19]. As generalists, grey seals likely forage on locally abundant prey [20] and accordingly, their diet may vary over both spatial and temporal scales [21–25].

Variations in prey assemblages over a range of spatial scales have been confirmed across grey seals’ distribution in the north Atlantic [23, 26–29]. However, studies on the diet of grey seals that haul-out along the coast of Ireland at the edge of the species range in the northeast Atlantic are outdated, temporally fragmented, and restricted in geographical range [30]. Biotelemetry studies conducted in the southeast and southwest of Ireland have demonstrated that grey seal foraging areas are centred around large haul-out locations, despite the potential for considerable movement between haul-out sites [16, 31]. These foraging areas, separated by a distance of approximately 300 km, represent two different habitats on the south-western Irish continental shelf, and the south-west Irish Sea.

Due to a combination of distinct oceanographic conditions [32, 33], the nutrient-rich waters surrounding Ireland experience high levels of surface productivity [34]. Considered internationally important commercial fishing grounds, these biologically productive waters also support many species of marine mammals [35, 36]. Against a backdrop of direct and indirect pressures on marine ecosystems [37], effective conservation of species and their habitat requires an understanding of the trophic interactions that drive the functioning of an ecosystem. Consequently, detailed regionally-specific data on predator diet for use in ecosystem models like Ecopath with Ecosim (EwE) are necessary. To make informed management decisions, the potential intra-specific regional variation in predator diet must be considered when implementing spatial plans over wider geographic scales.

Obtaining detailed information on seal diet composition at concurrent time scales, but from differing geographical areas, provides insights into prey community variability over relatively short distances. The primary aim of this study is therefore to investigate fine-scale spatial variation in the diet of grey seals from two contrasting ecosystems. Such high-resolution dietary information will provide insights into geographic variation in resource use, and can also be utilised in ecological modelling tools, thereby providing region-specific baseline data needed to more accurately reflect trophic pathways.

## Methods

### Ethics statement

Collection of scat samples for diet analysis was conducted without disturbing seals and as such required no ethical approval. All seal handling and tagging work was approved by University College Corks Animal Ethics Committee and conducted under licence from the Health Products Regulatory Authority (Project Authorisation AE19130/P004) and the National Parks and Wildlife Service, Ireland. The captured seals were restrained in hoop nets throughout the administration of the anaesthetic and prior to the tagging procedure. Seals were weighed to the nearest kg and anaesthetised using 0.05ml of Zoletil (Virbac; a combination of a dissociative anesthetic agent, tiletamine hypochloride, and a tranquilizer, zolazepamhypochloride) per 10kg delivered intravenously. Males were approximately 20% ‘under-drugged’ due to risk of entering deep dive reflex while under anaesthetic.

### Diet sample collection and analysis

Grey seal diet composition was compared using scat samples collected during the same period and by utilising sites that support high numbers of seals, thereby providing sufficient opportunity for scat collection. For robust baseline data, the use of the “all structures” method [38] to increase prey detection was employed. This method includes the identification of multiple diagnostic structures (e.g. premaxillae, vertebrae) in addition to fish otoliths and cephalopod beaks.

**Fig 1:**
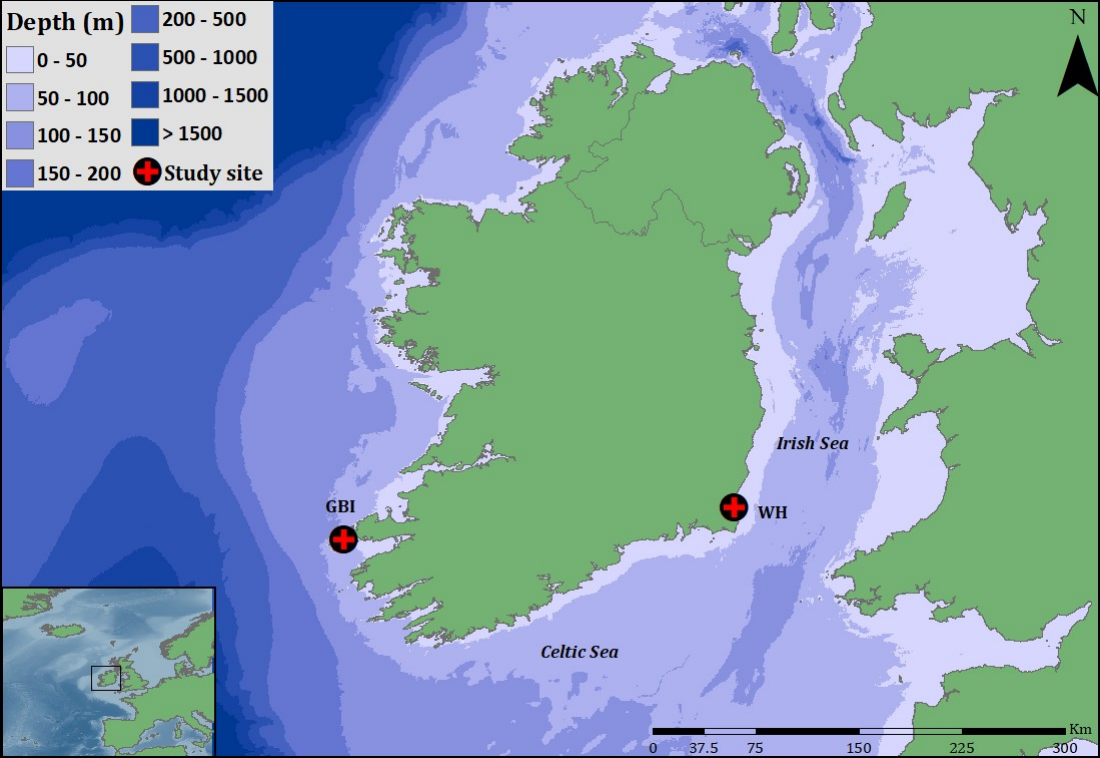
Study sites. Map of Ireland depicting surrounding water depth and study sites. Great Blasket Island (GBI) located off the southwest coast and Wexford Harbour (WH) on the southeast coast of Ireland.

Scat samples were collected from two nationally important grey seal breeding and moulting sites: Great Blasket Island (GBI) is located off the southwest coast of Ireland in the northeast Atlantic; and Wexford harbour (WH) is located on the southeast coast, in the Irish Sea (Fig 1). GBI contains the second largest grey seal breeding colony in Ireland, and supports a mixed colony of sexes and age groups with an all-age population size of between 1,099 and 1,413 individuals [39]. Seal numbers at WH range from less than 50 during winter months to 780 in summer months [31], with the site also supporting mixed ages and sexes. Sampling took place from August 2011 to November 2013, with haul-out sites accessed by boat when weather permitted, at or just after low tide. Scat samples from GBI were collected monthly (when conditions allowed access), while samples from WH were obtained on a bimonthly basis for the first 12 months and on a monthly basis thereafter. All samples were kept in labelled polythene bags and stored frozen at -20°C until further processing.

Organic material was separated from prey by washing individual scats through nested sieves (mesh size of 0.25 mm, 1 mm, 2 mm, and 5 mm) or by placing scats in nylon mesh bags and running them through two washing machine cycles, following Orr et al. [40]. Using the “all structures” approach [38], otoliths were corrected for partial erosion using grade-specific (when available) or species-specific digestion coefficients (see [41]). The minimum number of prey per scat was determined by counting the highest numbers of paired structures present (e.g., left or right otoliths/beaks/premaxillae etc.) or individual unique diagnostic structures such as an urohyal or vertebrae atlas (e.g., Ammodytidae). Multiple structures possibly originating from the same prey individual were matched according to colour and degree of erosion, and then through biomass reconstruction. Diet quantification was conservatively estimated to avoid duplication of prey items, (e.g., one ray, Rajidae, per presence of denticles) and biomass was reconstructed from otoliths as opposed to bones when present. Crustacean remains were excluded from dietary analysis as, due to their small size and poor condition, they were deemed to be secondarily ingested prey (cf. [42, 43]). Prey items were combined into guilds according to where in the water column they mainly occur based on the literature. Pelagic species were defined as those living in open waters occurring in the near-surface layers, while fish that live near the bottom, as well as in the midwater or near the surface, were defined as bentho-pelagic species [44, 45]. Benthic prey was divided into two groups: demersal fish were those occurring close to the bottom, while groundfish were defined as those occurring on or in the substrate [45, 46]. Finally, diet composition was expressed in terms of percentage frequency of occurrence (%F), percentage by number (%N), and percentage by biomass (%B), as described in Pierce and Boyle [47].

### Diet variability

A modified Costello feeding diagram [48, 49] was built to examine potential variability in grey seal diet between regions. The two-dimensional explanatory graph characterises diet variability by plotting the parameter prey-specific abundance (*P*_*i*_), as defined by Amundsen et al. [49], against the frequency of occurrence (F_*i*_).

Prey-specific abundance is expressed by:

$$P_{i}=\left(\frac{\sum S_{i}}{\sum S_{ti}}\right)\times 100$$

*P*_*i*_ represents the prey-specific abundance of prey *i*, S_i_ signifies the total contribution of prey *i* to the scat content, and S_ti_ denotes the total abundance of all prey within all samples containing prey *i*.

Frequency of occurrence is expressed as:

Fi=(NiN)×100

Where N*i* is the sum of all samples containing prey *i* and N is the total number of scat samples containing all prey within the diet.

Regional variation in consumed prey assemblages was also investigated using a multivariate approach in PRIMER 6 [50]. Samples were grouped into seasons within each year (Spring: February–April; Summer: May–July; Autumn: August–October; Winter: November–January), with prey species analysed according to their guild (pelagic, bentho-pelagic, demersal, groundfish). Square-root transformed species total abundances were used to generate a Bray-Curtis dissimilarity matrix in order to quantify dissimilarities between prey assemblages.

Bray-Curtis dissimilarity is defined as:

$$BC_{ij}=1-\frac{2C_{ji}}{S_{i}+S_{j}}$$

Where C*ij* signifies the total number of lesser values from species common at both sites, and S*i* and S*j* are the total number of specimens at each site.

A non-metric multidimensional scaling (nMDS) ordination plot was used for the visual representation of the Bray-Curtis resemblance matrix ranked data (refer to [51]). Each sample is represented by a symbol with the relative distance between samples representing their (dis)similarity to each other in terms of prey assemblages. As the plot is a 2D representation of the 3D graphical configurations derived from the ranked data in the Bray-Curtis resemblance matrix, the distance between symbols is not often apparent on visual inspection. Therefore, goodness-of-fit is represented by a *stress* value, which measures how well the inter-point distances in the 2D plots represent the rank-ordered inter-sample dissimilarities in the original matrix. Low *stress* values of ≤0.2 are considered good representations of the multi-dimensional data [50].

Analysis of similarities (ANOSIM, [51]) operating on the resemblance matrix outputs was used to test for differences in species groups between sampling sites within seasons and across years. The SIMPER routine in PRIMER 6 was then applied to the relative abundances of all guilds identified at both sites to determine which guilds, if any, were responsible for the greatest dissimilarities between regions. The analysis decomposes average Bray-Curtis dissimilarities between all pairs of samples (i.e. between sites) into percentage contributions from each species group, listing the groups in decreasing order of their contribution [51].

### Seal foraging area characterisation

We used satellite telemetry to determine the foraging distribution of adult grey seals at both sampling sites. Seal capture and deployment of Fastloc GPS/GSM tags was conducted at GBI in February to March 2009, 2011, and 2012, and at WH in March 2013 and 2014 (see Table 1). The fur was dried using paper towels and degreased with acetone, and the GPS/GSM tag (Sea Mammal Research Unit St Andrews University, full specifications available at http://www.smru.st-and.ac.uk/Instrumentation/downloads/GPS_Phone_Tag22.pdf) was then secured to the back of the neck using either fast setting epoxy resin (RS components) or superglue (Loctite). Tagging was conducted post-moult to maximise the period of attachment. Tags were programmed to attempt a location fix every 30 minutes and were only successful when coinciding with the animal being at the water surface. Once within range of the coastal GSM zone, the tags use GSM technology to relay data ashore via a data link call.

**Table 1:** Number of tagged seals.

Year	Site	Females	Males	Total
2009	GBI	8	0	8
2011	GBI	0	3	3
2012	GBI	0	3	3
2013	WH	1	4	5
2014	WH	3	6	9

The number of male and female grey seals tagged at GBI from 2009–2011, and WH from 2013–2014.

Minimum convex polygons (MCPs) were used to determine foraging areas most likely associated with scat samples collected at the two sites. GPS positions from tagged seals were plotted in ArcMap (ArcGIS version 10.4.1, ERSI, 2018), and projected to the ‘IRENET95 UTM Zone 29’ coordinate system. Locations within 1km of the haul-out sites were removed to account for intensive space use associated with seals coming and going from the sites or resting on exposed sandbars nearby, which may not be associated with foraging events. All fixes which occurred over land were similarly removed, as were any coordinates with readings either blank or latitude/longitude positions of zeros. The remaining points were given northings and eastings using the add XY coordinates tool. These were then used to create a 50% MCP using the MCP range tool from the ArcMET 10.4.1 extension (http://www.movementecology.net/arcmet_software.html). The percentage occurrence of sediment types within the 50% MCP was calculated for each sampling site. The outputted polygons were used to clip substrate data to the area of interest, then converted to raster layers and subsequently converted to points. These point layers were then used to extract depth values from a bathymetry raster layer. Sediment and depth data were obtained from the EMODnet portal. Sediment data were derived utilising output of the 2016 EUSeaMap broad-scale predictive model, produced by EMODnet Seabed Habitats (http://www.emodnet-seabedhabitats.eu). Depth data were downloaded from the EMODnet-bathymetry portal (http://portal.emodnet-bathymetry.eu/) as an ESRI ascii file.

## Results

### Diet analysis

A total of 355 scats containing prey were analysed with a minimum of 55 prey taxa identified across both sampling sites (Table 2).

**Table 2:** Scat collection effort.

Season	*GBI*				*WH*			
	2011	2012	2013	Total	2011	2012	2013	Total
Spring	-	21	20	**41**	-	19	29	**48**
Summer	-	20	20	**40**	-	21	8	**29**
Autumn	20	31	20	**71**	8	22	7	**37**
Winter	22	15	17	**54**	14	5	16	**35**
**Total**	**42**	**87**	**77**	**206**	**22**	**67**	**60**	**149**

Number of scats containing prey collected per site within each season across years.

### GBI diet composition

From 206 scat samples, 5,488 individual prey items, representing a minimum of 46 prey taxa were identified. The diet consisted predominately of teleost fish (100%F, 99%N, 97%B) (Table 3) of which gadoids were most important in terms of occurrence (84%F) and biomass (58%B). Amongst these, *Trisopterus* spp. (72%F, 13%N, 9%B) and haddock/pollock/saithe (*Melanogrammus aeglefinus/Pollachius pollachius/P*. *virens*) were the main contributors to the diet (29%F, 2%N, 24%B). However, sandeels (Ammodytidae) were numerically dominant and were both frequently occurring and substantial biomass contributors (61%F, 72%N, 17%B). Other prey species displaying relatively high dietary indices consisted of flatfish (43%F, 3%N, 13%B), blue whiting (*Micromesistius poutassou*) (25%F, 2%N, 3%B), Cephalopoda (23%F, 1%N, 3%B), and ling (*Molva molva*) (12%F, 1%N, 12%B). Overall bentho-pelagic species, including pollock/saithe, *Trisopterus* spp. and sandeel, occurred in the highest frequencies, were numerically dominant, and were the greatest biomass contributors to diet in this region (94%F, 87%N, 44%B).

**Table 3:** Grey seal diet composition.

Species	*GBI*			*WH*		
	%F	%N	%B	%F	%N	%B
PELAGIC	49.0	3.5	8.1	9.4	1.1	1.6
Herring *Clupea harengus*	2.9	0.1	0.71	2.7	0.6	1.50
Sprat *Sprattus sprattus*	10.2	0.4	0.02	4.0	0.3	0.05
Twait shad *Alosa fallax*	0.5	<0.1	0.03	-	-	-
Unidentified Clupeidae	1.9	0.1	0.06	2.0	0.1	0.06
Blue whiting *Micromesistius poutassou*	24.8	1.6	2.83	-	-	-
Silvery pout *Gadiculus argenteus*	1.5	0.1	<0.01	-	-	-
Garfish *Belone belone*	6.3	0.3	1.71	-	-	-
Horse mackerel *Trachurus trachurus*	14.1	0.7	2.48	-	-	-
Mackerel *Scomber scomber*	5.8	0.2	0.24	0.7	<0.1	0.01
BENTHO-PELAGIC	93.7	87.2	43.8	76.5	41.8	32.0
Pollock/Saithe *Pollachius* spp.	15.5	1.1	11.94	9.4	1.3	9.95
Whiting *Merlangius merlangus*	21.4	1.3	5.12	49.7	10.1	9.93
Norway pout *Trisopterus esmarkii*	4.4	0.2	0.03	-	-	-
Poor cod *Trisopterus minutus*	56.8	9.2	5.61	24.2	9.2	5.65
Bib *Trisopterus luscus*	14.6	0.8	2.59	3.4	0.3	1.16
Poor cod/Bib	9.7	0.7	0.70	6.0	0.6	0.82
Unidentified *Trisopterus* spp.	19.4	2.2	0.46	32.2	6.6	2.90
Greater forkbeard *Phycis blennoides*	1.0	0.1	0.16	0.7	0.1	0.02
Sea Breams Unidentified Sparidae	-	-	-	0.7	<0.1	<0.01
Greater sandeel *Hyperoplus lanceolatus*	16.0	3.2	4.10	7.4	3.5	0.85
Sandeels *Ammodytes spp*.	55.8	68.7	13.07	21.5	10.1	0.76
DEMERSAL	33.0	2.4	9.6	41.6	6.7	13.8
Cod *Gadus morhua*	1.5	0.1	0.72	6.7	0.7	2.32
Haddock *Melanogrammus aeglefinus*	10.7	0.6	4.26	4.0	0.6	1.70
Hake *Merluccius merluccius*	1.9	0.1	0.34	0.7	<0.1	0.19
Cuckoo wrasse *Labrus mixtus*	1.0	<0.1	0.02	-	-	-
Ballan wrasse *Labrus bergylta*	1.5	0.1	0.84	1.3	0.3	1.73
Unidentified Labridae	2.4	0.1	0.15	2.0	0.1	0.15
Squid *Loligo* spp.	5.8	0.2	<0.01	8.1	0.7	1.04
Squid Unidentified Ommastrephidae	3.9	0.1	0.02	5.4	0.6	0.54
Curled octopus *Eledone* spp.	3.4	0.2	0.66	9.4	1.2	1.95
Unidentified octopus	5.8	0.3	1.19	4.7	1.1	2.76
Unidentified Cephalopoda	10.7	0.6	1.42	16.8	1.3	1.39
GROUNDFISH	56.8	5.7	27.5	83.2	46.4	42.3
Ray *Raja* spp.	0.5	<0.1	0.03	42.3	2.7	2.42
Eels Anguilliformes	0.5	<0.1	<0.01	1.3	0.1	<0.01
Conger eel *Conger conger*	3.9	0.2	1.71	2.0	0.1	0.78
3-bearded rockling *Gaidropsarus vulgaris*	0.5	<0.1	0.05	2.0	0.6	0.67
4-bearded rocking *Rhinonemus cimbrius*	-	-	-	0.7	0.1	0.01
5-bearded rockling *Ciliata mustela*	1.0	0.1	0.02	-	-	-
Northern rockling *Ciliata septentrionalis*	-	-	-	4.7	0.4	0.25
Unidentified rocklings	1.0	<0.1	0.01	4.7	1.8	1.92
Ling *Molva molva*	11.7	0.5	11.68	0.7	<0.1	0.51
Dragonet *Callionymus spp*.	20.9	1.1	0.72	49.7	12.9	5.37
Grey gurnard *Eutrigla gurnardus*	4.9	0.3	0.66	8.1	1.0	2.36
Unidentified Triglidae	-	-	-	1.3	0.7	0.63
Shorthorn sculpin *Myoxocephalus scorpius*	-	-	-	1.3	0.1	0.25
Longspined bullhead *Taurulus bubalis*	-	-	-	2.0	0.2	0.10
Unidentified sculpins	0.5	<0.1	0.01	3.4	0.4	0.20
Unidentified Cottidae	1.0	<0.1	0.10	-	-	-
Pogge *Agonus cataphractus*	-	-	-	0.7	0.1	0.02
Eelpout *Zoarces viviparus*	-	-	-	0.7	<0.1	0.02
Butterfish *Pholis gunnellus*	-	-	-	7.4	0.9	0.15
Greater weever *Trachinus draco*	-	-	-	4.0	5.6	1.92
Tompot blenny *Parablennius gattorugine*	0.5	<0.1	-	-	-	-
Black goby *Gobius niger*	-	-	-	3.4	0.5	0.02
Painted goby *Pomatoschistus pictus*	-	-	-	0.7	<0.1	<0.01
Unidentified Gobiidae	0.5	<0.1	0.01	2.7	0.5	0.03
Megrim *Lepidorhombus* spp.	18.4	0.9	2.83	2.0	0.2	0.47
Scaldfish *Arnoglossus* spp.	5.3	0.2	0.12	-	-	-
Unidentified Bothidae	0.5	<0.1	0.02	2.0	0.2	0.50
Plaice *Pleuronectes platessa*	6.3	0.2	0.71	22.1	2.6	4.57
Dab *Limanda limanda*	3.9	0.2	0.55	13.4	3.1	1.50
Flounder *Platichthys flesus*	2.9	0.1	0.21	8.1	1.6	0.67
Plaice/Flounder	-	-	-	5.4	1.1	1.97
Dab/Flounder	-	-	-	1.3	0.1	0.12
Lemon sole *Microstomus kitt*	10.7	0.5	1.26	5.4	0.4	0.69
Long rough dab *Hippoglossoides platessoides*	1.5	0.1	0.26	4.7	0.3	0.18
Dab/Long rough dab	-	-	-	2.7	0.6	0.71
Witch *Glyptocephalus cynoglossus*	0.5	<0.1	0.06	0.7	<0.1	0.03
Unidentified Pleuronectidae	1.9	0.1	1.57	9.4	2.3	6.74
Solenette *Buglossidium luteum*	1.9	0.1	0.08	9.4	0.8	0.22
Sole *Solea solea*	2.4	0.1	0.76	14.8	1.1	3.36
Unidentified Soleidae	6.3	0.5	3.59	7.4	0.8	1.84
Unidentified flatfish	6.3	0.3	0.51	14.1	2.2	1.11
OTHER	19.4	1.3	11.0	30.2	4.1	10.3
Lamprey *Petromyzon marinus*	1.0	<0.1	-	0.7	<0.1	-
Haddock/*Pollachius* spp.	5.8	0.5	7.38	6.7	1.3	6.49
Unidentified Gadidae	12.1	0.7	3.59	23.5	2.5	3.81
Unidentified fish	3.9	0.1	-	3.4	0.2	-

%F = percentage frequency of occurrence, %N = percentage by number, %B = percentage biomass.

### WH diet composition

A total of 2,375 individual prey items, representing a minimum of 49 taxa, were identified from the 149 samples collected at WH. Cephalopod contribution to the diet in this region was higher than at GBI (35%F, 5%N, 8%B), while gadoids were the dominant component across all indices this time (85%F, 36%N, 48%B). Flatfish contribution to the diet was also substantially higher in WH (62%F, 18%N, 25%B) and of these, Pleuronectidae were by far the most important (45%F, 12%N, 17%B). Highest contributors within the Gadidae family similarly consisted of *Trisopterus* spp. (53%F, 17%N, 11%B) and haddock/pollock spp. (16%F, 3%N, 18%B), however whiting (*Merlangius merlangus*) (50%F, 10%N, 10%B) was of greater importance in this region compared with GBI. Other relatively important prey species included dragonets (*Callionymus* spp.) (50%F, 13%N, 5%B), rays (Rajidae) (42%F, 3%N, 2%B), and sandeels (26%F, 14%N, 2%B). In contrast to GBI, the diet at WH was dominated by groundfish species, including rays, dragonets, plaice (*Pleuronectes platessa*) and sole (*Solea solea*), with this guild accounting for the greatest proportion of the diet in this region (83%F, 46%N, 42%B).

### Diet variability

**Fig 2:**
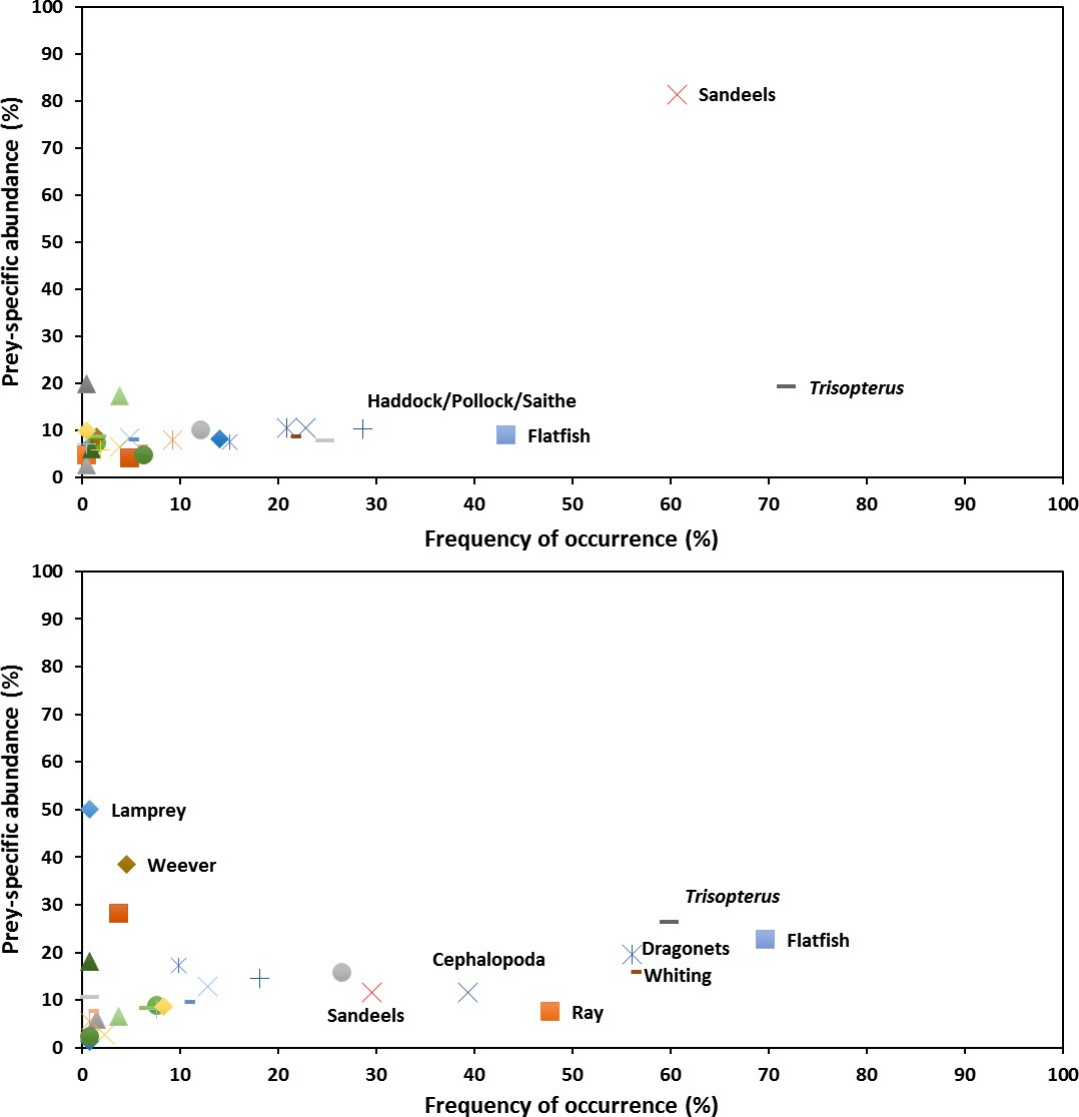
Modified Costello-Amundsen plots. Plots of all prey in terms of their occurrence and importance by number for GBI (upper frame) and WH (lower frame). Species occurring in the upper left represent prey that were consumed rarely, but when they were consumed, accounted for a large proportion of the predators’ diet. Species in the lower left denote prey that occurred rarely and were of relatively low importance to the overall diet. Species occurring in the upper right represent important prey found within the majority of diet samples that also accounted for a large part of the total diet. Finally, despite occurring in high frequencies, those prey species located towards the lower right corner of the diagram, only made a small contribution to the diet.

The majority of prey species in the diet from both sites were rarely observed in scat samples (< 15%F), and when present were typically observed in low proportions (*P* < 15%) (Fig 2). The Costello-Amundsen diagram highlighted sandeels as the dominant prey at GBI due to their frequent occurrence (> 60%F) and high proportions (*P* > 80%) within scat samples. *Trisopterus* spp. were regularly observed in scats (> 70%F), while haddock/pollock and flatfish were also common (> 25%F). However, all prey (apart from sandeels) were found in low quantities and thus accounted for only a small proportion of the overall diet (*P* < 20%).

Within scats collected from WH, no one prey species dominated the diet. Instead a number of prey (sandeels, cephalopods, rays, whiting, dragonets, *Trisopterus* spp. and flatfish) were highlighted as occurring often (30% < F < 70%), albeit in small proportions (*P* < 30%). Only lamprey (*Petromyzon marinus*) and greater weever fish (*Trachinus draco*) were responsible for a higher than average proportional contribution to the diet (*P* > 30%).

**Fig 3:**
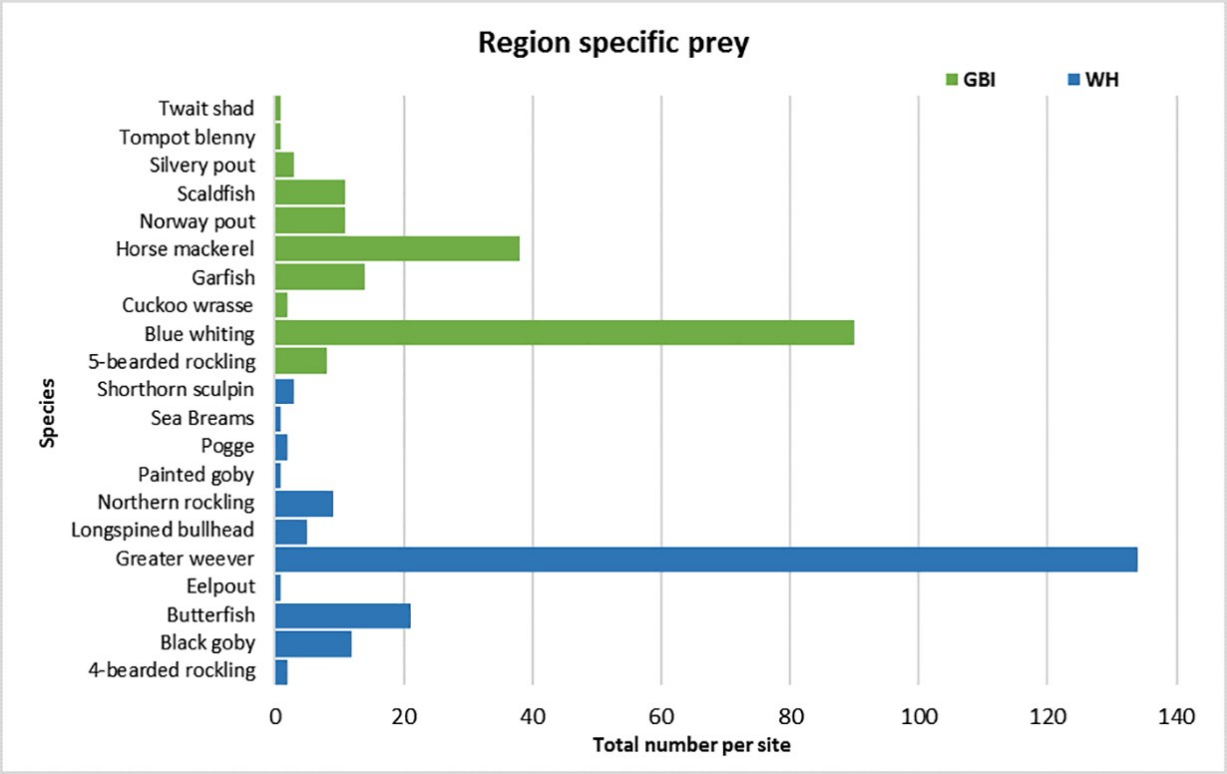
Region specific prey. The total numbers of prey species which occurred only in the southwest and in the southeast coast seal diet.

Certain prey species were unique to the diet from each region. Ten prey species, including pelagic fish such as blue whiting, horse mackerel (*Trachurus trachurus*), and garfish (*Belone belone*), were only found in the diet of seals hauling-out at GBI. Similarly, eleven species, including venomous weever fish and butterfish (*Pholis gunnellus*), only occurred in seal diet samples from WH (see Fig 3).

**Fig 4:**
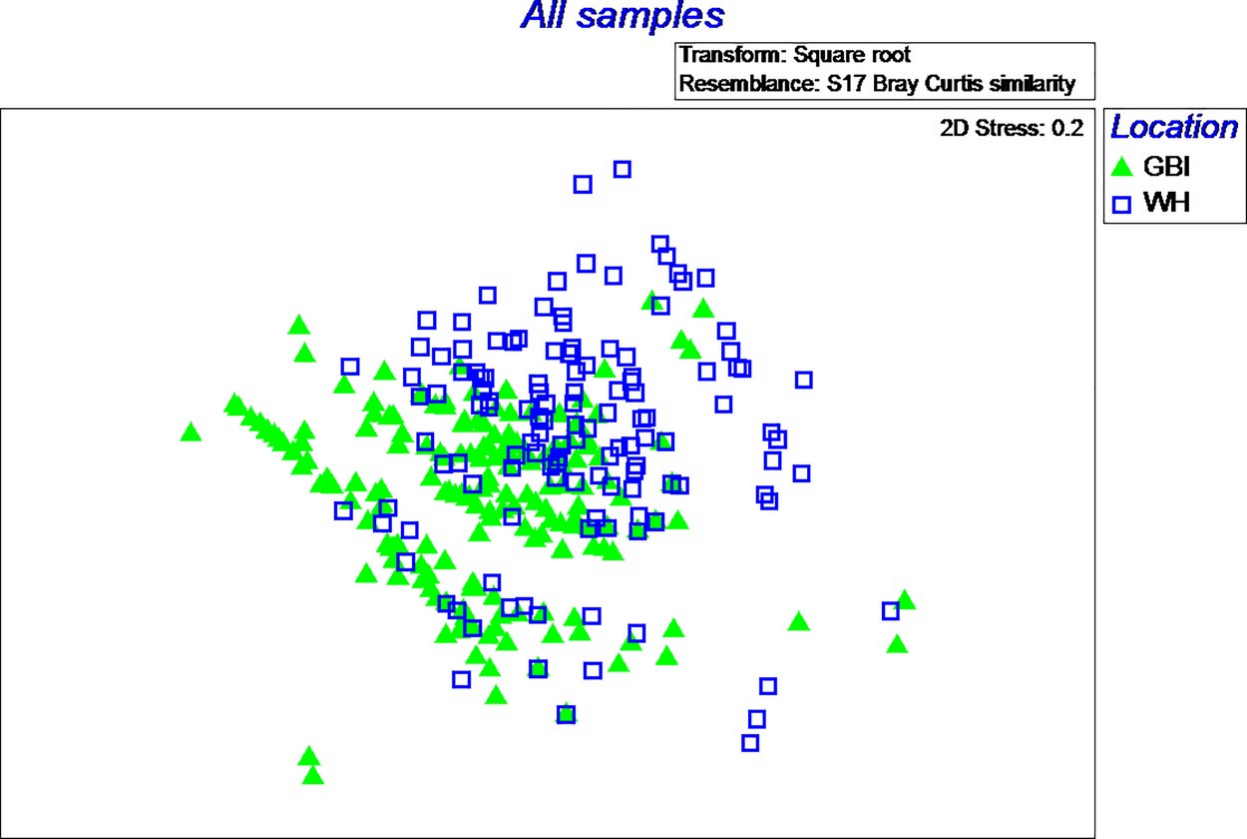
nMDS plot results. nMDS plot showing the multivariate patterns of prey species assemblages in all seal diet samples between each site. Each symbol represents an individual scat sample, with the relative distance between symbols representing (Bray-Curtis) similarity of prey assemblages (species and species abundance) between samples. The greater the relative distance the larger the dissimilarity between prey composition. The *stress* value of 0.2 suggests the data are a reasonable representation of the 3D structure.

To ascertain how similar species assemblages were between sites, a 2-D nMDS plot was produced (Fig 4). Samples from each site largely grouped together, with WH prey clustering above those from GBI, although some overlap in prey assemblages between sites was apparent.

To investigate whether prey assemblages differed between sites within season and across years, ANOSIM tests were conducted. The ANOSIM analysis confirmed significant differences in species guilds between sites in all seasons and all years (P≤0.005), apart from in winter (Table 4). Differences in the relative abundance of bentho-pelagic and groundfish prey consumed by seals were highlighted by the SIMPER analysis as accounting for the greatest dissimilarities in diet between sites (39% dissimilarity and 28% dissimilarity, respectively), with higher relative abundances of bentho-pelagics at GBI and higher relative abundances of groundfish at WH (Table 5).

**Table 4:** ANOSIM results.

Groups	Species guilds	
	*R statistic*	*P-value*
Spring	0.178	0.001
Summer	0.245	0.001
Autumn	0.304	0.001
Winter	0.048	0.055
2011	0.153	0.005
2012	0.186	0.001
2013	0.173	0.001

Results highlighting significant differences between GBI and WH in (square-root transformed) abundance data within species guilds (pelagic, bentho-pelagic, demersal, groundfish).

**Table 5:** SIMPER analysis results.

Groups	Species guilds		
	*GBI Average Abundance*	*WH Average Abundance*	*% Contribution*
Bentho-pelagic	3.37	1.85	38.92
Groundfish	0.87	2.08	27.72
Demersal	0.44	0.61	12.31
Pelagic	0.64	0.12	11.52
Other	0.24	0.40	9.54

Species guilds that contributed to the greatest dissimilarity between sites. % Contribution refers to the percentage each guild contributed towards the dissimilarity observed between sites.

**Fig 5:**
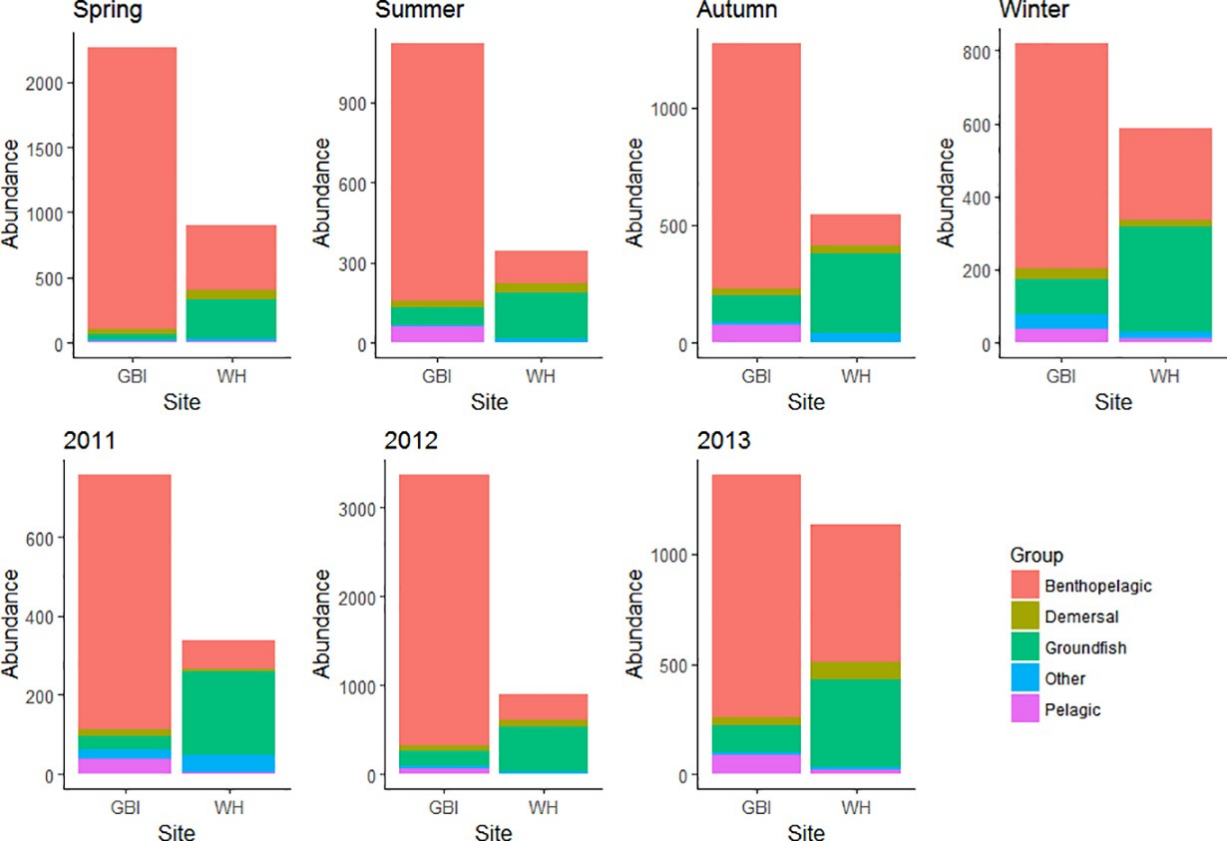
Total prey abundances. The total abundances of each guild detected in diet samples collected from both sampling sites across all seasons and all years.

The total abundances of each guild to the diet composition at both sites are displayed in Fig 5. During winter, far fewer prey items were consumed by seals at GBI, compared to that observed in other seasons. While prey guild abundances across all years varied significantly between sites, differences were less pronounced in 2011 (see Table 4).

### Telemetry analysis

**Fig 6:**
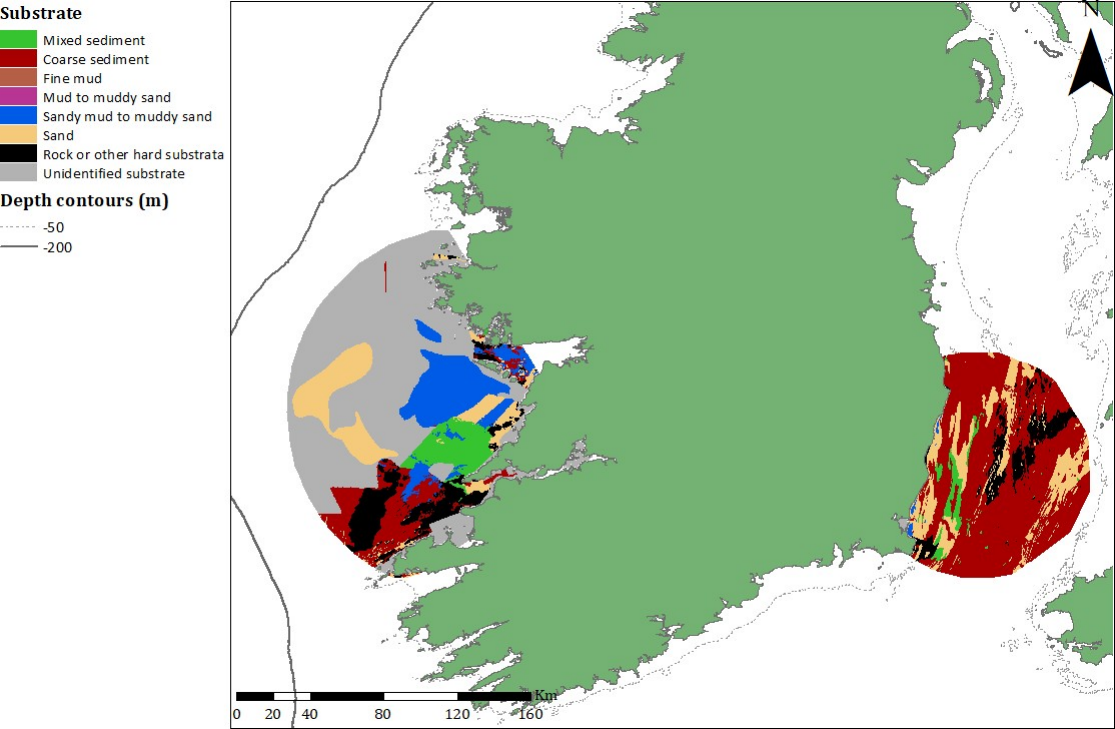
Minimum convex polygon results. MCPs containing 50% of seal GPS locations for grey seals tagged at GBI and WH. MCPs were superimposed over sediment data obtained for the EMODnet portal.

Telemetry data from 14 seals tagged at GBI and 14 seals at WH were utilised in this study (see Table 1). High site fidelity occurred at both tagging locations, but seals at GBI travelled further than those tagged at WH (Fig 6).

Seals tagged at GBI used a core area (50% MCP) along the west coast of Ireland (Fig 6). The core use area was limited to the shelf waters within the 200m depth contour. Much of the underlying sediment off the west coast of Ireland remains unclassified, particularly within the 50% MCP (55%). However, it is reasonable to assume that proportions of unclassified sediment are relatively consistent with the wider surrounding areas where sediment has been characterised. Excluding unknown sediment, within the 50% MCP, sandy mud/muddy sand (27%) and sand (24%) were the most commonly occurring substrates (Table 6). Seals tagged at WH utilised most of the Irish Sea, with the 50% MCP showing core foraging areas directly surrounding the WH study site (Fig 6). WH seals did not travel as far south as GBI seals, predominantly restricting foraging activity to the Irish and eastern-most part of the Celtic sea. Within the 50% MCP, coarse substrate (67%) and sand (19%) were the most common substrate types. The MCP at WH suggests seals in this region foraged in shallower water depths, with 78% of the MCP comprising depths of less than 90 m, whereas 70% of the MCP at GBI occurred in depths of greater than 90 m.

**Table 6:** Sediment type and water depth at each sampling site.

Habitat variable		Great Blasket Island (GBI)	Wexford Harbour (WH)
**Sediment**	Coarse sediment	18.5%	67.0%
	Sandy mud to muddy sand	26.8%	0.4%
	Sand	24.2%	19.2%
	Rock or other hard substrata	18.3%	9.8%
	Mixed sediment	12.2%	3.6%
**Depth**	0–30	8.8%	14.6%
	30–60	9.1%	12.1%
	60–90	12.5%	51.3%
	90–120	41.7%	21.7%
	120–150	27.9%	0.3%

50% MCP representing core foraging habitat of seals tagged at GBI and at WH (excluding unknown substrate).

## Discussion

Foraging strategies and diet can vary greatly within species and populations [14, 26, 27, 52]. As grey seals act as central place foragers, their diet likely reflects the available prey adjacent to haul-outs. In this study, grey seals displayed significant regional variation in their diet, despite the common occurrence of key prey (e.g. *Trisopterus*, sandeels, flatfish and Cephalopoda). Grey seals at GBI are more frequent consumers of bentho-pelagic prey such as sandeels, *Trisopterus* and pollock species. They also consume greater quantities of pelagic prey with a number of species identified in the diet completely absent from samples collected at WH (e.g. blue whiting, horse mackerel, silvery pout (*Gadiculus argenteus*), and garfish). In contrast, groundfish abundance was over eight times higher in diet samples from WH, with prey such as rays, dragonets and sole also occurring in substantially higher frequencies. A number of other groundfish (e.g. weever fish), were exclusive to grey seal diet in this region, while demersal cephalopods were also more prevalent in samples analysed from this haul-out site. The high frequencies of rays (likely thornback rays, *Raja clavata*) within the diet of seals foraging around WH has not previously been documented.

Temporal variation in diet was apparent with significant differences in diet noted between sites in all years and seasons except winter. This may be due to seals at GBI consuming fewer prey in winter, as noted in previous studies where grey seals switch from small species like sandeels during the summer to larger gadoids in winter [26, 53]. Regional variation in prey guilds were less apparent in 2011, and are likely to be influenced by lower sample sizes in 2011 where scat collection only occurred in autumn and winter. Temporal variation in diet is likely due to a combination of factors associated with prey availability. Prey assemblages will differ over time due to variations in distribution that are symptomatic of a changing biological community governed by natural forces such as the primary productivity, spawning stock biomass, recruitment, spawning and timing of migration. Climate change has also been linked to changes in fish abundances in Irish waters [54].

The differing energy requirements of grey seals during their annual life cycle may similarly contribute to the temporal and spatial variations observed in the diet, with the age and sex structure of a population likely changing between sites over the course of a year. Indeed, both sites support highly variable intra-annual populations [16, 31]. Grey seals exhibit strong individual variation in their foraging habitat [55] and ontogenetic differences in prey preferences are also known to exist [29] with juveniles tending to be less selective, exhibiting a broader niche breath [25]. Furthermore, while grey seals are considered to be central place foragers, they regularly move between haul-out locations, and are capable of spending extended periods at sea [16, 17]. As scat samples can represent the previous 2–3 days meals [41], it is possible that scats collected at sampling sites may not represent local prey species occurring within the 50% MCPs. Additionally, while we tracked both male and female seals from each site, tracking data were limited to adult seals which, may not fully reflect the foraging range or habitat of juvenile seals. While Carter et al. [56] found that juvenile grey seals spent substantially longer offshore without returning to the coast in the North Sea, juveniles in the Celtic and Irish Seas remained within 50 km of their natal haul-out sites. Furthermore, stable isotope analysis suggests juveniles rely more on carbon sources derived from areas relatively closer to shore [57]. Within this study, 50% MCPs of telemetry data from both sites indicate core foraging areas in close proximity of haul-out sites, which is in agreement with previous telemetry studies which found that seals generally forage within 50 km of their haul-out sites [16, 17, 31].

We make the reasonable assumption that scat content in this study is representative of mixed seal populations supporting all ages and sexes. Scat samples were collected concurrently from both locations, and while scat collection did not always coincide with the timing of the tagging studies, overlap did occur, and the multi-year tagging provides for inter-annual variation in foraging areas such that diet content is likely to be reflective of spatial use derived from the telemetry data. While it is not possible to infer specialisation from scat content, it is likely that seals at each site constitute a collection of generalist predators comprised of a number of specialist individuals. Given the age and sex-specific differences in dietary requirements, life history and foraging strategies, scat contents may represent different cohorts/sexes depending on where and when they were collected, and do not necessarily represent the diet composition of individuals over time. Prey which were highly prevalent in the seals diet at each location are among the most abundantly recorded species in those areas. For example, *Trisopterus* spp. and blue whiting are abundant in the Celtic Sea [58], while members of the Pleuronectidae family are known to dominate fish assemblages in the Irish Sea [59].

Fish community assemblages are correlated with seabed/sediment type and the structural complexity of their habitat [60–62]. The differing habitats and prey availability surrounding both haul-out sites are likely contributors to the significant spatial variation in relative prey assemblages observed between sites. The predominant sediment type surrounding GBI consists of sand, with large pockets of rock and coarse sediment located directly north of the haul-out site. While much of the sediment off the west coast of Ireland remains unclassified, habitat data inferred from fisheries assessments suggest large pockets of mud and sand comprise seabed substrate in this region (T. Keena, pers comm.). In contrast, the Irish Sea is dominated by coarse sediments, with sediment type surrounding WH consisting of sand and smaller pockets of rock. Water depth is likely to influence prey selection, as deep water may limit the time available to forage at the benthos. Close to 95% of oxygen is stored in the blood and muscle of grey seals [63]. During deep dives, a large proportion of these reserves are depleted in transit to and from the benthos [1], with seals often restricting their swimming activity or remaining motionless on the bottom [64]. Furthermore, seals with free access to surface water have been shown to preferentially select short dives that do not extend beyond their aerobic dive limit (ADL) capability estimates [65]. These factors have implications for seal foraging activity, and are potential contributors to the differences in prey assemblages observed in this study.

Grey seals tagged at GBI tended to utilise considerably deeper water (>90 m), over predominantly sand and sandy/mud substrate, which could account for the substantial quantities of pelagic species and sandeels observed in the diet [66]. Waters covering coarse sediment and rock were also important areas of core utilisation and the presence of this sediment type within close range of the haul-out site may explain the occurrence of whiting, wrasse (Labridae spp.), conger, and ling observed in the diet of seals in this region [45] as these species are associated with coarse sediment and rocky habitat. The higher prevalence of pelagic species in diet samples is also likely related to these prey being more readily available in this region, given its proximity to the shelf edge and open water. This is also consistent with dive behaviour of seals tagged at GBI, with 31% of dives being pelagic and occurring more frequently over mud and sandy sediments [67]. The deeper waters off GBI may furthermore allow for comparatively less foraging time along the seabed.

Prey species displaying the highest prevalence in diet samples obtained from WH similarly appear to be directly associated with the substrate type in the Irish Sea. Grey seals in this region spent substantially more time utilising shallower waters (<90 m) with coarse sediment dominating core use areas. High frequencies of whiting in the diet can be attributed to the coarser sediments that prevail throughout the Irish Sea, with their presence also well documented given the large whiting fishery that exists in this region [68]. Seals also utilised more areas characterised by sand, which is consistent with a recent study where grey seals satellite tagged at WH were found to forage over mud and sand [69]. Plaice, dab (*Limanda limanda*), sole, dragonets, weever fish and rays were amongst the most frequently occurring prey identified in this region, all of which are associated with sandy bottoms [45, 59]. Groundfish assemblages, particularly plaice and dab, are known to dominate the shallower waters of the Irish Sea [59, 70]. Foraging in these relatively shallower waters would enable seals to spend more time at the benthos while still remaining within their ADL, facilitating the large presence of benthic species in the diet.

The waters surrounding Ireland contain some of the most valuable commercial fisheries resources in Europe, with an estimated landed value of €1.44 billion in 2017 [71]. The results of this study indicate seal predation on commercially important fish populations at regional scales that requires further attention. Pelagic species like blue whiting and horse mackerel are of substantial economic importance and both occur frequently in the diet of seals utilising waters of west and southwest Ireland. As these are also migratory species, their prevalence in seal diets requires consideration by fisheries managers. Similarly, the high frequencies of rays and plaice, commonly targeted by commercial fisheries in the Irish and eastern Celtic Seas, occurring in the diet of grey seals utilising these waters also highlights potential seal-fishery conflict in this region. It is likely that the contribution of rays to the diet in this study is underrepresented, as abundance was conservatively estimated at one ray per scat when denticles were present. Furthermore, a conservative average length of 29.5 cm [45] was assigned to each individual ray within the diet based on harbour seals (*Phoca vitulina*) consuming predominantly juveniles rays [43]. Data from annual Irish groundfish surveys suggest a length of 54 cm is common along the coast of Ireland for this species [72]. Should grey seals be consuming non-juvenile *R*. *clavata* at 54 cm, this would make them equally as important to the diet as flatfish, in terms of biomass contribution.

While Gosch et al. [38] highlighted significant temporal variation in the diet of grey seals off southwest Ireland, this study has demonstrated significant spatial/regional differences in prey species, likely linked to habitat. Grey seals utilising deeper waters over predominantly sandy bottoms close to the continental shelf break likely experience higher encounter rates with blue whiting and sandeels, and consequently consume more of these pelagic and bentho-pelagic prey. In contrast, seals utilising the comparatively shallower waters of the Irish/eastern Celtic Seas are greater consumers of benthic species, possibly as a result of greater foraging time at the benthos. Sediment types found in this region also facilitate the high prevalence of groundfish in the diet, such as plaice and sole. The high frequency of certain economically valuable prey species warrants further investigation into the potential for greater resource conflict occurring in these regions. Variability in grey seal diet, both within and between regions, also has important implications for ecosystem models based on generic trophic pathways, as these are likely too broad a tool for effective ecosystem management. Rather than assuming predator diet is consistent across ecosystems, this study emphasises the importance of incorporating regional diet information when constructing fisheries and ecosystem-based models.

## Supporting information

S1 Appendix Prey abundances, frequencies, weights, and seal ID and tracking locations. (XLSX)
